# From Cave Dragons to Genomics: Advancements in the Study of Subterranean Tetrapods

**DOI:** 10.1093/biosci/biab117

**Published:** 2021-12-08

**Authors:** Hans Recknagel, Peter Trontelj

**Affiliations:** University of Ljubljana, Slovenia, working, Biotechnical Faculty, Dept. of Biology, Subterranean Biology Lab; University of Ljubljana, Slovenia, working, Biotechnical Faculty, Dept. of Biology, Subterranean Biology Lab

**Keywords:** cave adaptation, troglomorphism, salamanders, selection, biomedicine

## Abstract

Throughout most of the kingdom Animalia, evolutionary transitions from surface life to a life permanently bound to caves and other subterranean habitats have occurred innumerous times. Not so in tetrapods, where a mere 14 cave-obligate species—all plethodontid and proteid salamanders—are known. We discuss why cave tetrapods are so exceptional and why only salamanders have made the transition. Their evolution follows predictable and convergent, albeit independent pathways. Among the many known changes associated with transitions to subterranean life, eye degeneration, starvation resistance, and longevity are especially relevant to human biomedical research. Recently, sequences of salamander genomes have become available opening up genomic research for cave tetrapods. We discuss new genomic methods that can spur our understanding of the evolutionary mechanisms behind convergent phenotypic change, the relative roles of selective and neutral evolution, cryptic species diversity, and data relevant for conservation such as effective population size and demography.

Caves and other subterranean environments host a large biodiversity with more than 50,000 species obligately bound to these habitats around the world (Culver and Pipan 2019). Among them, tetrapods constitute a negligible fraction but have historically shaped the views and misconceptions about life in caves. From medieval fictional dragons to Darwin's (1859) speculation about the eyes of cave rats (*Neotoma*) changing by use and disuse, it has always been cave tetrapods that have fascinated people more than other subterranean life forms. From paleontological finds in caves, species such as the cave bear (*Ursus spelaeus* Rosenmüller, 1794), cave lion (*Panthera spelaea* Goldfuss, 1810), and cave hyena (*Crocuta crocuta spelaea* Goldfuss, 1823) have been described. Their names are reminiscent of the mythical perception of large and dangerous animals lurking deep underground to maul the unfortunate lost wanderer. The truth about cave tetrapods is perhaps less dramatic but just as exciting and mysterious.

To begin with, we should clarify that if an animal uses caves as shelter or den, this does not make it a cave species. Our focus is on those species that have adapted to lives in the subterranean realm to the degree that makes them unfit for life on the surface. We use the term *cave* in a wide sense that includes smaller crevices, as well as water-filled subterranean spaces. In general, a species is considered a cave obligate if it is not found to voluntarily move outside the cave—for example, at night or in wet weather—and if it completes its entire life cycle, from embryonic development to reproduction and death, exclusively within caves. In this review, we focus on cave-obligate tetrapods, which have rarely been reviewed as a whole, probably because of their inaccessibility, scattered distribution, and extreme endemism and rarity (figure 1; for reviews, see Weber 2000, Gorički et al. 2019, Soares and Niemiller 2020). The latter two factors are largely because of the paucity of evolutionary transitions from surface to cave life in tetrapods. It is currently unclear why this is the case, given that the transition has occurred thousands of times in invertebrates and hundreds of times in fishes (Mohr and Poulson 1966, Hüppop 2000, Culver and Pipan 2019).

**Figure 1.**
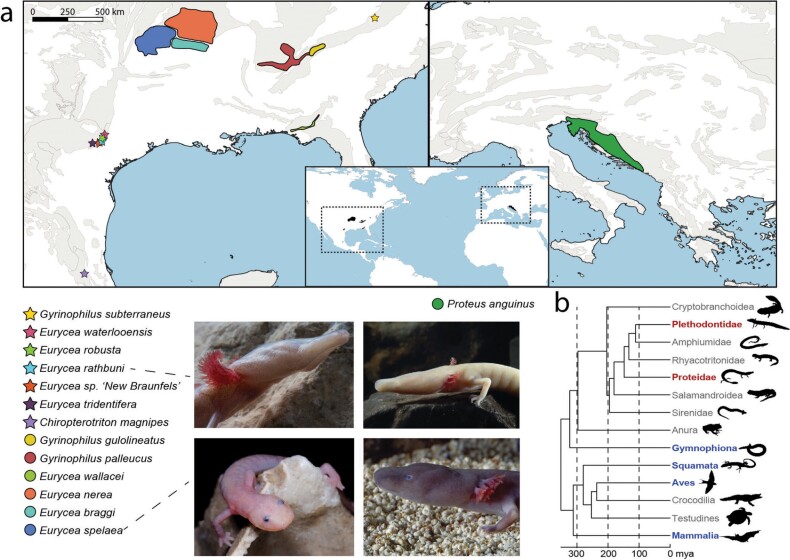
Global distribution of obligate cave tetrapods. Karstic areas are marked in light grey. (a) Several North American salamander species are known from single or few locations with very confined ranges (marked with stars). (b) Phylogeny with molecular divergence times of major tetrapod groups that evolved cave obligate species (in bold red), groups that include species that occasionally dwell in caves or below ground (in bold blue), and groups that are not found in caves or other subterranean habitats (in light grey). Abbreviation: mya, million years ago. Photographs: Dante Fenolio (Eurycea spelaea, Eurycea rathbuni), Arne Hodalič (Proteus anguinus, Proteus anguinus parkelj).

To understand why cave tetrapods are so exceptional, a short general introduction to the ecology and evolution of cave life is needed. Although the subterranean environment may seem hostile for organisms adapted to life on the surface, a myriad of species across the kingdom Animalia have adapted to a life in caves. Here, organisms are exposed to an environment abounding with abiotic and biotic extremes. Abiotic conditions often include a complete lack of light, high humidity, a bare and rocky substrate, a lack of circannual and circadian rhythms, and sometimes high levels of radiation from radon (Moldovan et al. 2018). Within the ecosystem, organic production is low or absent; so are nutrients in any form of organic carbon but also competitors and predators. These conditions are so drastically different from the conditions experienced at the surface that most animals do not establish intergenerational populations in caves (Gibert and Deharveng 2002, Moldovan et al. 2018, Culver and Pipan 2019). Those that do, often exhibit, to some extent, preadapted characters, such as a fossorial or nocturnal life history or a preference for humid and dark places (Wilkens and Strecker 2017, Culver and Pipan 2019, Howarth 2019). The abiotic and biotic conditions in caves pose various evolutionary challenges that promote evolutionary change in the colonizing populations. At the level of morphology and life history, these changes are highly predictable (traits by which cave species differ from their surface counterparts are called *troglomorphisms*; box 1, figure 1) and include traits that are reduced (regressive traits) and others that are elaborated or enhanced (constructive traits).

**Box 1. Troglomorphic traits.**

Obligate cave-dwelling animals display a suite of characteristic traits called *troglomorphisms* (Christiansen 2012). These are generally independently evolved and shared across taxonomic groups, but can differ on a few morphological and physiological characteristics that are unique to specific groups (e.g., elongation of appendages is typical of arthropods; Moldovan et al. 2018). Traits can also be antagonistic; for example, some cave salamanders evolved longer limbs, whereas, in others, limbs are reduced. Troglomorphisms can be subdivided into two main categories, traits that are reduced (regressive traits) and traits that show modification (constructive traits) relative to surface ancestors. Lists of nonmorphological traits are not exhaustive, and more research is likely to reveal other traits typical for cave-obligate tetrapods. See table 1.

**Table 1.** Troglomorphic traits.

	Constructive traits	Regressive traits
Morphology	Head or limb elongation and flattening, olfactory system, inner ear, taste buds, lateral line system	Eye loss, depigmentation, limb reduction, digit loss, reproductive anomalies
Physiology	Starvation resistance	Weakened circadian rhythm, lower metabolic rate
Behavior	Feeding habits	Loss of aggressive behavior or complex social behaviors
Life history	Increased offspring size, longevity	Reduced clutch or litter size

Ever since Darwin, the evolutionary processes leading to these outcomes have been a source of controversy among scientists. Relaxed selection, random drift of traits no longer under selection, directional selection or a combination thereof may have led to the observed patterns, in particular for reduced traits that exhibit a loss of function (Rétaux and Casane 2013). Research on the Mexican cavefish *Astyanax mexicanus* has greatly advanced our understanding of the evolutionary and genetic causes of phenotypic change in cave vertebrates, supporting both adaptive and nonadaptive forces leading to the evolution of reduced traits (Culver and Pipan 2015, Casane and Rétaux 2016, Gross et al. 2016, Cartwright et al. 2017, Krishnan and Rohner 2017). Although *Astyanax* cavefish are an extraordinary model species for cave biologists, they also represent a particular case with evolutionarily young lineages, which is not necessarily representative for many obligate cave vertebrates.

Understanding the biology of obligate cave tetrapods can help resolve various contemporary issues, from conservation to genome evolution and medicine. For example, in the light of recent discoveries highlighting the biomedical relevance of cave species (Riddle et al. 2018, Jeffery 2020), it is paramount to include cave-obligate tetrapods as humans’ closest living relatives in caves. Known traits in cave-dwelling vertebrates that may be of particular biomedical relevance for humans include longevity, circadian rhythms, eye development, and resistance to starvation and obesity, as well as recently discovered reproductive and cytogenetic anomalies (Sessions et al. 2016, Bizjak-Mali 2017).

## Cave-obligate tetrapods: The number of species and transitions from surface to caves

Salamanders of the families Plethodontidae and Proteidae are the only tetrapods that evolved cave-obligate species. The true number of species is unclear and in flux, with 14 cave-obligate species currently reported (table 2), but this is likely to be an underestimate because of cryptic species (Gorički and Trontelj 2006, Trontelj et al. 2007, Bendik et al. 2013, Phillips et al. 2017, Devitt et al. 2019, Gorički et al. 2019, Corbin 2020). Cave-obligate plethodontids and proteids share several troglomorphic traits and show a high degree of convergence both within and between families (table 3, figure 1).

**Table 2.** List of cave-obligate salamander species.

Species	Common name	Authority	IUCN status	Degree of endemism
*Chiropterotriton magnipes*	Big-footed salamander	Rabb 1965	Endangered	Several sites
*Eurycea rathbuni*	Texas blind salamander	Stejneger 1896	Vulnerable	Few sites
*Eurycea robusta*	Blanco blind salamander	Longley 1978	Data deficient	Single site
*Eurycea waterlooensis*	Austin blind salamander	Hillis et al. 2001	Vulnerable	Single site
*Eurycea braggi*	Southern grotto salamander	Smith 1968	NA	Region
*Eurycea nerea*	Northern grotto salamander	Bishop 1944	NA	Region
*Eurycea spelaea*	Western grotto salamander	Stejneger 1892	Least concern	Region
*Eurycea wallacei*	Georgia blind salamander	Carr 1939	Vulnerable	Several sites
*Eurycea tridentifera*	Comal blind salamander	Mitchell and Reddall 1965	Vulnerable	Several sites
*Eurycea sp. ‘New Braunfels’*	NA	mentioned in Goricˇki et al. (2019)	NA	Single site
*Gyrinophilus palleucus*	Tennessee cave salamander	McCrady 1954	Vulnerable	Region
*Gyrinophilus gulolineatus*	Berry cave salamander	Brandon 1965	Endangered	Region
*Gyrinophilus subterraneus*	West Virginia spring salamander	Besharse and Holsinger 1977	Endangered	Single cave
*Proteus anguinus*	Olm, Proteus	Laurenti 1768	Vulnerable	Region

**Table 3.** List of troglomorphic features in cave-obligate salamanders.

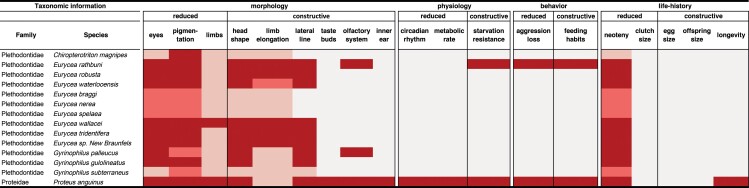

*Note:* All species belong to the order of salamanders. The colors describe the approximate degree of troglomorphism: Dark red represents troglomorphic, light red represents weakly troglomorphic, pink represents nontroglomorphic, and light grey represents no data available.

The obligate subterranean Plethodontidae (lungless salamanders) are restricted to North America. They occasionally share the subterranean habitat with several facultative cave species of the same family, particularly in the genus *Eurycea*. They form both cave and surface populations that present a challenge for species delimitation (Bendik et al. 2013, Phillips et al. 2017, Devitt et al. 2019). The 13 currently reported cave-obligate plethodontid salamanders exhibit varying degrees of troglomorphisms. Eye loss, pigmentation loss and paedomorphosis are common to almost all of them with a few exceptions (table 3). The level of troglomorphism differs among species, and some facultatively surface-dwelling species also exhibit some troglomorphic traits (e.g., Bendik et al. 2013). The differences in troglomorphism may allow plethodontid salamanders to exploit different niches within the cave environment. Adaptation to subterranean micro niches and diversification within caves has been shown also for invertebrate fauna (Borko et al. 2021).

Although paedomorphosis is not a troglomorphic trait and shared with several surface-dwelling amphibians, it may be involved in the evolution of other troglomorphic traits, such as head morphology, enhanced lateral line system and taste buds (Gorički et al. 2019). The correlation between paedomorphosis and cave dwelling in salamanders is well known. It has been suggested that the constant, nutrient-poor subterranean environment favors paedomorphosis. One way nutrients can enter caves is via infiltrating surface water and sinking rivers, making the aquatic part of the subterranean environment on average less nutrient deprived than the terrestrial one. Paedomorphic salamanders are permanently aquatic and, as such, have access to a richer and denser assortment of prey (Brandon 1971, Wilbur and Collins 1973, Bruce 1979). Paedomorphosis can arise by two mechanisms: neoteny, which is the delay of metamorphosis, or progenesis, which is accelerated sexual maturation. In North American cave salamanders, species of the genus *Eurycea* probably derived from an ancestor that was able to skip metamorphosis by early maturation (Ryan and Bruce 2000). Therefore, these species are believed to avoid metamorphosis by progenesis. In contrast, in the group of *Gyrinophilus* cave salamanders, neoteny seems to be the mechanism driving paedomorphosis (Bruce 1979) and has presumably evolved after cave colonization (Ryan and Bruce 2000). Within the plethodontid cave-obligate species, the grotto salamanders (*Eurycea spelaea*, *Eurycea nerea*, *Eurycea braggi*) are the only salamanders that regularly undergo metamorphosis acquiring typical terrestrial traits (see figure 1). *Gyrinophilus subterraneus* undergoes metamorphosis at an extremely large size (Besharse and Holsinger 1977, Niemiller et al. 2009), but it is unclear how regularly this occurs and how long the terrestrial form survives. In addition, the Mexican *Chiropterotriton magnipes*, the southernmost cave salamander, is fully terrestrial. Whether this species represents a true obligate cave dweller is questionable as it may move outside caves to find new subterranean habitats and has large eyes (Capshaw et al. 2019). A relict plethodontid lineage from mainland Italy and Sardinia is known as the European cave salamanders (genus *Speleomantes*). Although they are regularly found in caves, they spend part of their life outside of caves and are not troglomorphic (Ficetola et al. 2018).

Proteidae occur in North America and Europe, but have evolved cave-obligate forms only in Southeastern Europe. North American mudpuppies (*Necturus*) inhabit surface fresh waters, whereas the European sister genus *Proteus* is represented by a single nominal cave-obligate species, the olm (*Proteus anguinus*). Neoteny is a conserved trait of the family present already in the common surface ancestor. *Proteus anguinus* was the first cave-obligate species described to the scientific community although it was probably not recognized as such by its describer, Laurenti (1768). Although most *Proteus anguinus* populations show several constructive as well as regressive troglomorphic traits, such as degenerate eyes, reduced pigmentation, digit reduction, body elongation, an elaborated lateral line system, and an elongated snout with various kinds of receptors, a less troglomorphic, darker (but neotenic) form with normally developed eyes has been discovered relatively recently (Sket and Arntzen 1994). Interestingly, this lineage stems from the youngest split within the mitochondrial phylogeny of geographically separated highly troglomorphic lineages (Gorički and Trontelj 2006). This suggests either that multiple independent invasions to caves occurred from surface populations that are now extinct or the more parsimonious scenario of a localized, evolutionary reversal of a troglomorphic to nontroglomorphic phenotype (Ivanović et al. 2013, Sessions et al. 2015). Moreover, the old splitting times (molecular estimates reach more than 10 million years back) between *Proteus* lineages, absence of detectable gene flow and morphological differences between them suggest several cryptic species within this group (Trontelj et al. 2009, Gorički et al. 2017).

The number of identified cave-obligate salamanders is likely to increase in the near future, albeit not dramatically. Discoveries of new species can be expected mostly among known taxa through better understanding of gene flow boundaries and will be facilitated by the use of genome-wide molecular markers in combination with novel taxonomic approaches. For many cave salamanders, the degree and variation of various troglomorphic traits is still unknown (table 3). In addition to the classical troglomorphic traits, other physiological, life history, reproductive, and behavioral traits may also be modified relative to their surface ancestors, but again, this is largely unknown at present.

## Why are there so few cave-obligate tetrapods?

Given the high number of taxonomic groups that have successfully and independently evolved specialized cave species in invertebrates (Deharveng and Bedos 2018) and ray-finned fishes (Moldovan 2018, Soares and Niemiller 2020), it may come as a surprise that no other tetrapod group except salamanders has made the transition. Explanations as to why this might be so include endothermy (Mohr and Poulson 1966) and terrestriality (Hüppop 1985) coupled with the low nutrient availability in caves. Although in aquatic caves food input via sinking rivers is possible, terrestrial subterranean habitats are energetically much more deprived. Mammals and birds, being endotherm organisms, require large amounts of energy to maintain their body temperature and are at a large disadvantage. Even for a shrew-size mammal, the endotherm metabolism consumes more energy than the animal could possibly ingest in such nutrient limited environments (Mohr and Poulson 1966). Some endotherm species partially rely on caves as habitat, including numerous species of bats, rodents (e.g., various rat-size species colloquially referred to as *cave rats*, dormice *Glis glis*), oilbirds (*Steatornis caripensis*), and swiftlets (chiefly genera *Aerodramus* and *Collocalia*). These use caves for breeding, as well as roosts and hibernacula, whereas all foraging takes place outside the cave. In contrast, reptiles—as energetically highly efficient ectotherms—may be able to cope with the low food availability in caves. The nocturnal lifestyle of tropical night lizards (genus *Lepidophyma*) could constitute a preadaptation in the context of the darkness experienced in caves, and it has been suggested that some species have reduced pigmentation (Smith and del Toro 1977). However, other factors make full transitions to a cave life in reptiles unlikely, including their strong dependence on external heat sources and almost exclusively terrestrial lifestyle (Weber 2000).

In addition, some tetrapods have evolved a completely fossorial lifestyle in the deep soil where they inhabit preexistent burrows or burrows they dig themselves (box 2). In this organically rich habitat they feed either as herbivores on plant roots or as predators on a myriad of soil invertebrates. These species can share some of the characteristic traits found in obligate cave species. This includes fossorial rodents (Begall et al. 2007); many fossorial squamates, including both lizards and snakes (Sites et al. 2011); and caecilians (Wake 1985). For example, the naked mole rat (*Heterocephalus glaber*) is a eusocial mammal that has evolved reduced and constructive traits also typical for obligate cave species, such as a partial reduction of eyes and pigmentation, enhanced tactile sensory organs, longevity, and a slow metabolism. Similarly, fossorial blind snakes (mainly the families Typhlopidae, Xenotyphlopidae and Anomalepididae) and worm lizards (Amphisbaenia) have evolved reduced eyes and pigmentation (box 2). Traits associated with a fossorial lifestyle may make them more suited for cave life than their surface-dwelling relatives, but no tetrapod species has made the switch from a fossorial to a cave environment. The main reason may be that the soil still provides enough surface-derived nutrients, whereas caves are severely nutrient restricted.

**Box 2. Fossorial tetrapods.**

**Figure 2.**
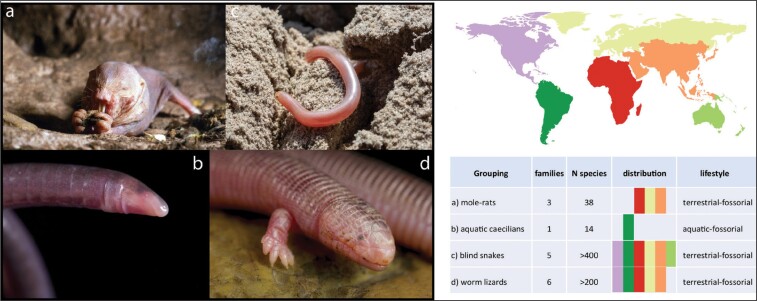
The colors on the map refer to continents and are represented in the table as presence of a group in the respective continental region. Photographs: (a) *Heterocephalus glaber*, Neil Bromhall/ Shutterstock.com; (b) *Typhlonectes compressicauda* and (d) *Bipes canaliculatis*, reptiles4all/Shutterstock.com; (c) *Rhinotyphlops lalandei*, Willem Van Zyl/Shutterstock.com.

Subterranean fossorial tetrapods convergently evolved traits shared with obligate cave-dwellers. These include regressive traits such as reduction of eyes and pigmentation (figure 2a–2d), and constructive traits such as longevity and enhanced tactile sensory organs (figure 2a). A fossorial lifestyle has evolved repeatedly in mammals (figure 2a), caecilians (figure 2b), snakes (figure 2c) and lizards (figure 2d). Within these groups, several fossorial species have evolved across the globe. However, despite the phenotypic similarities no fossorial tetrapod has made the transition from a soil-dwelling to an obligate cave-dwelling lifestyle. The reason for this may be the contrast in nutrient availability: Although the soil receives many nutrients from leaf litter, roots, microorganisms and invertebrates, caves are more nutrient poor. Tetrapods—and especially endotherms—require large amounts of energy to maintain their bodily functions, a physiology that is challenged by the low nutrient availability in caves. Other primarily fossorial tetrapods include some salamanders (e.g., Oedipina) and frogs (e.g., *Neobatrachus*), that have, however, not evolved the typical traits shared with cave dwellers.

Finally, it seems clear that, in contrast to all other tetrapods, the amphibian physiology predisposes some groups to evolve cave forms. However, even in these groups lineage-specific biases are obvious: Only salamanders and, within these, only the plethodontid and proteid lineages were able to make the transition (Weber 2000, Gorički et al. 2019). Why did frogs and caecilians or even other salamander families not evolve obligate cave forms? Caecilians are adapted to subterranean habitats and, much like cave-adapted animals, have degenerated eyes (Wake 1985). However, the burrowing lifestyle may be of limited use in the cave environment characterized by solid and rocky substratum; terrestrial forms are therefore unlikely to evolve. Only the lungless, aquatic family Typhlonectidae from South America (San Mauro et al. 2014) seems to have potential to evolve cave forms. Nevertheless, no such caecilians have been observed in any of the numerous Brazilian tropical caves (Rodrigo Lopes Ferreira, Universidade Federal de Lavras, Minas Gerais, Brazil, personal communication, 5 January 2021). Various frog species have been reported from caves, although usually they enter caves accidentally (Biswas 2010) or only temporarily during certain developmental stages (Diesel et al. 1995), to hibernate or aestivate, whereas foraging takes place outside the cave (Joglar et al. 1996).

A few key traits emerge that may predispose lungless and proteid salamanders to evolve cave-obligate species: an ectotherm, energy-saving metabolism; a tendency toward permanent aquatic life and paedomorphosis; small body size in plethodontids; nocturnal, scotophilic lifestyle coupled with poor vision in proteids (Gorički et al. 2019). Nevertheless, predicting whether a lineage is predestined to evolve cave forms or not remains a challenge (Ribera et al. 2018, Culver and Pipan 2019). This is illustrated by both families possessing traits that seem incompatible with a subterranean life. Many adult terrestrial plethodontid salamanders hunt using a projectile tongue and have excellent vison, traits that are of little use in caves. Although some plethodontids are nocturnally active and do not have projectile tongues, including the occasionally cave-dwelling *Eurycea lucifuga* (e.g., Hutton et al. 2019), only paedomorphic, aquatic forms have made it to obligate cave dwellers. The sole exception that regularly undergoes full metamorphosis in caves is the grotto salamander *Eurycea spelaea*. Members of this species exhibit an omnivorous diet (Soares et al. 2017) and sometimes live in association with bat colonies, where they can feed on the dense invertebrate fauna and even on the nutrient-rich bat guano itself (Fenolio et al. 2014). On the other hand, *Proteus* has inherited its aquatic and darkness-loving habits from surface ancestors. Likewise, it has inherited its large body reaching close to 0.1 kilogram, which is about an order of magnitude above the mass of most North American cave salamanders but comparable to the size of the largest known individuals of the Berry Cave salamander (*Gyrinophilus gulolineatus*; Gladstone et al. 2018). Sustaining a body of this size seems to be in conflict with the energy-poor subterranean ecosystem. A possible explanation lies in the biological richness of some subterranean waters of the Dinaric Karst that are home to *Proteus*, and in the high organic input from the surface in the Berry Cave (Gladstone et al. 2018).

## What is the genetic basis of traits related to cave life in tetrapods?

It is of great interest to evolutionary biologists to understand how organisms evolved troglomorphic traits—that is, which genes are responsible for these traits (Pardo-Diaz et al. 2015, O'Quin and McGaugh 2016, Wilkens and Strecker 2017). Cave-obligate animals represent one of the most prominent examples of convergent phenotypic evolution. The question remains whether convergence of troglomorphic traits is mirrored on the genetic level. In addition, this research has biomedical relevance, in particular understanding circadian rhythms, eye development and diseases, reproductive and cytogenetic anomalies, and resistance to starvation and obesity (Sessions et al. 2016, Riddle et al. 2018, Jeffery 2020).

In recent years, the genetic architecture and basis of several troglomorphic traits has been revealed through research on *Astyanax* cavefish, demonstrating that troglomorphisms can have a simple (e.g., pigmentation: Protas et al. 2006, Keene et al. 2015) or complex (e.g., eye sight: Casane and Rétaux 2016, Warren et al. 2021) genetic basis, as well as QTL affecting several troglomorphic traits (pleiotropy: Protas et al. 2008, Yoshizawa et al. 2012). Research on invertebrates has shown some commonality with the genetic basis for eye loss and pigmentation, although differences were also observed (Protas et al. 2011, Aspira et al. 2012, Protas and Jeffery 2012). For example, a single gene causes eye loss and multiple genes cause pigmentation loss in freshwater crustaceans *Asellus aquaticus* (Protas et al. 2011). Reduced traits appear to have evolved under the influence of genetic drift (Wilkens and Strecker 2017), whereas eye reduction may have been shaped at least in part by natural selection, as was suggested, for example, by the proximity of eye size QTLs to sites under selection in the genome (Borowsky 2015).

Both genetic architecture and the strength of selection influence how quickly traits evolve. If these variables differ among traits, the order in which traits appear after the colonization of caves should also differ on a temporal scale. Accordingly, research on *Astyanax* cavefish indicated that lineages that made the transition from surface to caves longer ago show genetically more complex changes (Wilkens and Strecker 2003), and eye reduction appears to occur more rapidly than genetically driven pigment loss despite its more complex genetic basis (Borowsky 2015). Furthermore, the variance of troglomorphic traits within a cave population or species also appears to differ with time since cave colonization. This is presumably because of the polygenic nature of troglomorphisms and the fact that causal mutations are not fixed in younger cave populations, resulting in a gradual range of phenotypes that depend on the number of troglomorphism-causing mutations. In summary, the research mostly focused on *Astyanax* cavefish has shown that troglomorphic traits are mostly polygenic, potentially arranged in clusters, and genetically independently derived in lineages that have independently colonized caves (Keene et al. 2015, Wilkens and Strecker 2017). The central role of particular developmentally important genes, such as *shh* and *pax6*, may constitute common genetic factors involved in troglomorphisms in vertebrates (Yamamoto et al. 2004, Jeffery 2019). Unfortunately, a comprehensive meta-analysis examining these relationships in more detail across a range of animals (or smaller groups such as tetrapods) is lacking but is urgently needed if we are to understand general principles on how troglomorphisms arise. For example, it remains to be determined how troglomorphic traits may be related to observed reproductive and cytogenetic anomalies in salamanders (Sessions et al. 2016, Bizjak-Mali 2017). This lack of alternative model systems is primarily because of biological constraints posed by long generation times, rarity, and low reproductive output of cave tetrapods. However, recent technological and methodological advances in genomic analyses can overcome some of these challenges in the near future. In the following, we will provide some suggestions on how this will be possible.

The first method of choice for identifying the genetic basis of phenotypic traits is QTL mapping (e.g., Casane and Rétaux 2016, O'Quin and McGaugh 2016). The greatest challenge faced by researchers—and particularly for those studying tetrapods—is the reliance on species that exhibit closely related surface and cave-dwelling lineages, and that are easy to breed in large numbers. Because these requirements are rarely fulfilled, QTL mapping renders most, if not all cave-obligate tetrapods with troglomorphic traits unsuitable for genetic research. The alternative method to uncover the genetic variation causing phenotypic variation is genome-wide association (GWAS) mapping. Unlike QTL mapping, this method does not require a known pedigree scheme but, instead, relies on the phenotypic variation in natural populations and historical recombination events (Wellenreuther and Hansson 2016). Many cave tetrapods may be suitable for applying GWAS on troglomorphisms: Particularly at early stages of evolution, mutations leading to troglomorphic traits have not been fixed in cave populations, and the phenotypic variability often by far exceeds those on the surface (Wilkens and Strecker 2017). In addition, cave populations experiencing gene flow with surface populations are also suited for this approach. For example, a study in *Gyrinophilus* cave-obligate salamanders showed that there has been recurrent gene flow with their surface-dwelling relative *Gyrinophilus porphyriticus* (Niemiller et al. 2008). Such cases are ideal for studying the genetic basis of troglomorphisms. However, the rarity and generally low densities of cave-obligate tetrapods observed in nature will remain a major challenge. Cave species are usually under strong conservation and protection, and obtaining tissue samples is often not justifiable.

QTL mapping and GWAS can be complemented with genetic analyses that do not measure phenotype-genotype correlations directly, such as genome scans; differential expression-based analyses, including RNA sequencing (RNASeq) and open chromatin sequencing (ATAC-Seq); differential methylation analysis (e.g., whole genome bisulfite sequencing); and analyzing protein-nucleotide interactions by chromatin immunoprecipitation sequencing (ChIP-seq). Genome scans rely on mechanisms of selection acting on the phenotypic variation of interest, which manifests itself as genetic differentiation between individuals expressing the distinct trait. This assumes that selected genetic variation can be differentiated from variation produced by neutral processes such as drift or population stratification, making this approach less powerful if not combined with other approaches (Wellenreuther and Hansson 2016). For example, a study across several *Astyanax* cavefish and surface populations revealed convergence in regions of differentiation between surface and cave populations, and these regions were linked to some previously identified QTLs for troglomorphic traits (Bradic et al. 2013). RNA-Seq, ATAC-Seq and ChIP-Seq have been successfully used to complement, confirm, and refine results from traditional GWAS and QTL mapping approaches (e.g., Nica et al. 2010, Banovich et al. 2014, Li et al. 2016, Bendesky et al. 2017). Considering that many troglomorphic traits may exhibit phenotypic plasticity (Bilandžija et al. 2020), approaches that incorporate this aspect are ideal.

Differential analyses based on single cells used in a complementary way can be further used to identify the functional basis of traits, in particular if rare cell types are responsible for a phenotype (Hendrickson et al. 2018, Jia et al. 2018, Liu and Montgomery 2020). For example, comparative single-cell sequencing of the mouse and naked mole-rat immune systems revealed some unique features in the naked mole rat's system that might be associated with its longevity and cancer resistance (Hilton et al. 2019). In addition, this method could prove useful to identify the particular cell types showing dysfunctional pathways in degenerated tissues such as eyes in obligate cave species.

Because many causal QTL are noncoding, genetic mapping approaches have often missed to identify the molecular function of QTLs (Do et al. 2017, Liu and Montgomery 2020). This gap is filled by mapping expression or methylation QTLs (eQTL and mQTL) across individuals, or using allele-specific expression methods within individuals (Gaur et al. 2013, Wang et al. 2020).

The drawbacks of many of these methods is the requirement of a reference genome, and high-quality tissues, in addition to financial resources. The large genomes of salamanders are an additional challenge, although methods targeting functional molecular variation such as RNA-Seq or ATAC-Seq should prove to yield comparable data to other vertebrates, although this remains to be explored. The sequencing of the axolotl (*Ambystoma mexicanum*; Nowoshilow et al. 2018), giant salamanders (Sun and Mueller 2014), Iberian ribbed newt (*Pleurodeles waltl*; Elewa et al. 2017), and olm (*Proteus anguinus*; Kostanjšek et al. 2021) genomes will prove useful resources and set cave salamanders up as new model organisms for studying the genetics of troglomorphisms and for biomedical research as the closest cave-adapted relatives to humans.

## Conservation of rare and cryptic cave-obligate tetrapods

Threats to cave life include habitat destruction (Elliott 2012, Furey and Racey 2015, Gallão and Bichuette 2018), aquifer overexploitation (Griebler et al. 2019), climate change (Mammola et al. 2019), pollution (du Preez et al. 2016, Gallão and Bichuette 2018), tourism (Ferreira et al. 2020), and transported diseases (Reynolds and Barton 2014, Li et al. 2020). A major threat to aquatic cave salamanders is the pollution and exploitation of groundwater aquifers and cave waters (Miller and Niemiller 2008, Pezdirc et al. 2011, Bendik et al. 2014, Ribeiro and Tičar 2017, Devitt et al. 2019). For example, *Eurycea* cave salamander species inhabiting the Edwards Aquifer have extremely small ranges and are therefore particularly threatened by the depletion of groundwater in this area (Chippindale and Price 2005, Devitt et al. 2019, Sharp et al. 2019). In Slovenia, olms are experiencing increased levels of pollutants in their environment (Năpăruș-Aljančič et al. 2017, Ribeiro and Tičar 2017) and these at least partially accumulate in their tissue (Pezdirc et al. 2011, Bizjak Mali and Bulog 2016). Another potential threat to cave salamanders are diseases. Amphibians are currently devastated by the chytrid fungus disease, which has led to a loss of diversity especially in America (Alroy 2015) and Europe (Martel et al. 2014, Stegen et al. 2017). In America and parts of Africa, the chytrid fungus *Batrachochytrium dendrobatidis* has resulted in heavy decline of anuran populations, whereas in Europe the spread of *Batrachochytrium salamandrivorans* has been devastating in particular to salamanders. As cave salamanders occur in these regions and may therefore be exposed to chytrid fungus, this is a major concern. Thus far, no cave salamander populations have tested positive for the fungus (Fenolio et al. 2013, Kostanjšek et al. 2019), and there are indications that *Proteus* salamanders show some tolerance to *B. salamandrivorans* (Li et al. 2020). However, the overall impact that the disease may have on natural populations is currently impossible to assess, and minimizing the potential for contact should be prioritized.

Efficient conservation of subterranean species requires reliable data on the size, distribution and connectivity of populations (see table 2). These are difficult to obtain for aquatic species that inhabit inaccessible karstic aquifers such as the Texas blind salamander (*Eurycea rathbuni*) from the Edwards Aquifer in Texas and several *Proteus* lineages in the Dinaric Karst. However, in cave regions accessible to researchers for detecting and counting individuals, population abundances and sizes have been assessed in *Gyrinophilus* salamanders (Miller and Niemiller 2008, Niemiller et al. 2010). Mark–recapture studies conducted over multiple years are ideal but can only rarely be done and are usually restricted to small local areas. Pilot studies were conducted for *Eurycea spelaea* (Fenolio et al. 2014), *Eurycea rathbuni* (Pierce et al. 2014) and for *Proteus anguinus* (Balázs et al. 2020). To avoid potential negative consequences to individuals induced by tagging, and gain insight into populations inaccessible to researchers, population size estimates derived from genetic data (usually estimated as effective population size, *N*e) by swabbing can in principle be employed (Luikart et al. 2010). In populations with panmixia or occasional admixture across the cave system, including the spaces inaccessible to humans, genomes will show diversity signatures indicative of long term population size in the entire area. By this approach, using microsatellite data, Zakšek and colleagues (2018) looked at the nearly panmictic *Proteus* population of the Postojna–Planina Cave System in Slovenia, which is probably one of the largest populations of any subterranean salamander. The link disequilibrium method (Waples and Do 2010) suggested that *N*e of this *Proteus* population was about 1100.

Demographic inference using site-frequency spectra (quantifying the relative frequency of alleles at different rarities present in a population) reliant on genome-wide data allows for the estimation of gene flow and effective population sizes across populations of interest (Marchi et al. 2021). These have been previously shown to lead to accurate population size estimates in simulations (Nunziata and Weisrock 2018) and empirically in salamanders (Nunziata et al. 2017), and are a promising tool for assessing population sizes of cryptic species.

Instead of relying on the diversity signatures imprinted into individual genomes, another possibility is the use of environmental DNA (eDNA; Thomsen and Willerslev 2015). Sampling the waters of subterranean systems can reveal the presence of sites previously unknown to harbor a particular species (Vörös et al. 2017, Boyd et al. 2020). The presence of *Proteus* in Montenegro has been long anticipated, and it was finally confirmed by eDNA survey (Gorički et al. 2017). In a quantitative sense, several studies have inferred total biomass of a species on the basis of eDNA quantity in experimental setups (Pilliod et al. 2013, Lacoursière-Roussel et al. 2016, Doi et al. 2017). This relationship is more complex in a natural environment (Cristescu and Hebert 2018, Yates et al. 2019). A multitude of factors, including environmental (e.g., pH, temperature) and biological (e.g., life-history traits) effects confound the relationship between eDNA quantity and biomass (Pilliod et al. 2014, Lacoursière-Roussel et al. 2016, Yates et al. 2019). For example, it is difficult to obtain reliable age structure estimates in a wild population, particularly if there are temporal fluctuations and a lag in eDNA distribution (Maruyama et al. 2014). In addition, there are methodological challenges, and procedures have not yet been fully standardized (Cristescu and Hebert 2018, Yates et al. 2019, Beng and Corlett 2020). Nonetheless, there is no doubt that eDNA quantification holds a promising future for estimating diversity and population size (Cristescu and Hebert 2018). It will prove even more powerful if coupled with next-generation sequencing techniques once prevailing challenges have been resolved (Adams et al. 2019, Sigsgaard et al. 2020).

## Conclusions

Obligate cave-dwelling tetrapods are an exceedingly rare phenomenon and are limited to salamanders. Despite their rarity, they hold promise as model organisms for the study of the evolution of complex vertebrate-specific traits changing in animals moving from surface to caves. Being closest to humans of all obligate cave organisms, they are of special interest for biomedical research of longevity, tissue and organ regeneration, skin and eye diseases, reproductive biology, obesity and starvation. Because salamanders have successfully evolved to cave life multiple times independently, they allow for a quantitative assessment on the ecological, physiological, evolutionary, developmental, and genetic mechanisms. Comparing the genetics of cave adaptation within and between the two deeply divergent lineages of cave-obligate Proteidae and Plethodontidae should provide particularly useful insights. Cave salamanders are of high conservation concern and require innovative approaches to conservation biology as their habitat is inaccessible to researchers but prone to pollution. This research will greatly benefit from new advances in sequencing technology and methodological advances. Although the large genomes of salamanders have previously been considered a barrier to such research, the recent and ongoing genome sequencing of salamanders removes this limitation, providing the resources to open this line of research to scientists with broad and diverse interests in evolutionary biology.

## Author Biographical

Hans Recknagel (hans.recknagel@bf.uni-lj.si) is an evolutionary biologist supported by a Marie Skłodowska-Curie Actions fellowship and affiliated with the University of Ljubljana, Slovenia, working at the Biotechnical Faculty, Dept. of Biology, Subterranean Biology Lab. Peter Trontelj is a professor of evolutionary biology at the University of Ljubljana, Slovenia, working at the Biotechnical Faculty, Dept. of Biology, Subterranean Biology Lab.
